# Dietary supplement and medication use in professional and pre-professional dancers: widespread use but limited evidence of benefit—a systematic review

**DOI:** 10.3389/fnut.2026.1850279

**Published:** 2026-06-04

**Authors:** Marian Vela-Andreu, Juan Jesús Montalvo-Alonso, Marta del Val-Manzano, José Alberto Martínez-Hortelano, Alberto Pérez-López

**Affiliations:** 1 Facultad de Medicina y Ciencias de la Salud, Departamento de Ciencias Biomédicas, Área de Educación Física y Deportiva, Universidad de Alcalá, Madrid, Spain; 2 Facultad de Enfermería, Universidad de Castilla-La Mancha, Albacete, Spain

**Keywords:** analgesic agents, dancers, dietary supplements, ergogenic substances, musculoskeletal pain, physical performance

## Abstract

**Introduction:**

Professional and pre-professional dancers experience exceptional physical demands and an increased risk of injuries and health concerns, often leading to the use of medication and dietary supplements.

**Objective:**

To examine the prevalence of dietary supplement and medication utilization among professional and pre-professional dancers and their effects on performance, health outcomes, and dance-related pathologies.

**Methods:**

This systematic review followed PRISMA 2020 guidelines and was registered in PROSPERO (CRD420251000872). PubMed, Scopus, Web of Science, and Cochrane Library were searched from inception to April 2025. Eligible studies included professional or pre-professional dancers using dietary supplements or medication with potential therapeutic or ergogenic effects. Data extraction and risk-of-bias assessments were performed independently by two reviewers using RoB 2, ROBINS-I, and JBI tools.

**Results:**

A total of 22 studies were included (12 observational, 6 randomized controlled and 4 non-randomized interventions) involving 654 female and 86 male dancers from various disciplines. Supplement use ranged from 11 to 57%, with multivitamins, vitamin C, caffeine, and isotonic beverages most frequently consumed. Vitamin D improved isometric strength, jump performance, and reduced injury incidence. Iron decreased anemia prevalence, while folic acid enhanced endothelial function. Whey protein and creatine increased lean mass without consistent performance benefits. Analgesics and non-steroidal anti-inflammatory medication were reported by up to 90% of dancers.

**Conclusion:**

Pharmacological and nutritional substances use among dancers is widespread yet heterogeneous, with limited evidence of benefits and potential health risks. High-quality randomized controlled trials are needed to establish evidence-based guidelines for safe and effective use in dance populations.

**Systematic review registration:**

https://www.crd.york.ac.uk/PROSPERO/view/CRD420251000872, CRD420251000872.

## 1 Introduction

Professional and pre-professional dancers represent a population with exceptional physical demands, combining high volumes of repetitive loading, extreme ranges of motion, coordination, and balance (1, 2). Training and performance schedules often exceed six hours per day, that together with the physical demands confer a distinctive injury and medical-care profile compared with other athletic groups (2, 3). Given such demanding schedules, dancers are particularly vulnerable to low energy availability (LEA) and fatigue from inadequate energy and micronutrient intake (4).

Musculoskeletal pain and physical complaints leading to difficulties participating in normal class, and missed rehearsal or performance time are common, resulting in a high incidence of injuries, especially overuse (5–7). Findings indicate that overall, the prevalence, incidence, and anatomical location of overuse injuries do not differ between male and female ballet and contemporary dancers (8, 9). Prospective studies in professional ballet dancers have reported injury incidence rates per 1,000 dance hours ranging from 3.9 to 4.14 in females and 3.1 to 4.76 in males, with overuse injuries accounting for 68% in females and 60% to 64.7% in males of all reported injuries (7, 10). The lower extremity is the most frequently affected anatomical region, particularly involving the foot, ankle and knee (8, 9, 11).

These clinical burdens often prompt the use of pharmaceuticals and nutraceuticals to manage symptoms, sustain function, or enhance performance and recovery (2, 5). A widespread use of dietary supplements in dance populations has been documented. Previous studies have reported a variable, moderate-to-high prevalence of dietary supplement use among dancers, with estimates ranging from 48% to over 90% across cohorts, for reasons such as maintaining good health, boosting immunity, reducing fatigue, improving energy, or preventing injury or illness (12, 13). Commonly reported products include caffeine, multivitamins and vitamin C, while protein supplements and vitamin D have also been described across cohorts (12).

Evidence on specific supplements suggests potential but limited benefit to dancers. Vitamin D insufficiency has been associated with increased risk of musculoskeletal injury, and interventional studies in a small population of dancers suggest potential reductions in injury incidence and enhanced muscle function with supplementation, although dancer-specific evidence remains scarce (14). Conversely, caffeine has a well-characterized, dose-dependent ergogenic effect on multiple domains of physical and cognitive performance outcomes in athletes (15), but its safety and optimal application in dance, where fine motor control, balance, and stage-related cardiovascular arousal are relevant, has not been comprehensively synthesized.

Epidemiological studies and questionnaires indicate that self-reported use of analgesics and non-steroidal anti-inflammatory medication (NSAID) is also frequent among dancers and athletes more broadly, prevalences ranging between 70% and 90% have been reported among dancers and other athletic populations (13, 16). Many studies reporting regular or prophylactic intake to control pain and enable continued participation as primary reasons (5, 6). This practice raises the concern of masking and delayed reporting of pain and injury, which may negatively impact injury severity, prognostics and treatment (6). Contemporary meta-analyses and systematic reviews in athletes found that NSAIDs do not reliably improve neuromuscular performance or recovery and their routine prophylactic use is not supported as an ergogenic strategy (17), even though recent evidence highlights the potential hypertrophy benefits (18).

Beyond musculoskeletal outcomes, the choreography of pharmacotherapy and dietary supplement use in dancer populations raises additional concerns including the potential between-substance interactions (i.e., pharmacokinetic and pharmacodynamic), psychoactive effects on coordination and proprioception, and the unregulated or poorly labelled composition of some dietary supplements, which may all affect safety and performance in ways that are not yet well quantified (19, 20).

Patterns of use among dancers, from therapeutic to misuse, underscore the need to understand not only prevalence but also motivations, sources of advice and the medical oversight surrounding these practices (21). Existing studies in dancers have primarily focused on prevalence and descriptive patterns of use, with comparatively few have evaluated physiological, performance-related, or health effects. Moreover, the available evidence is heterogeneous across dance disciplines, populations, and outcomes assessed, and findings regarding potential therapeutic or deleterious and ergogenic or ergolytic effects of these interventions in dancers remain incomplete. This fragmentation of the literature highlights the need for a comprehensive synthesis. Thus, this systematic review aims to identify the most prevalent dietary supplements and medications used among professional and pre-professional dancers across disciplines, and assess their effects on dance and physical performance, as well as on dance-related pathologies and injuries.

## 2 Methods

### 2.1 Registration and protocol

This systematic review was conducted according to the Preferred Reporting Items for Systematic Reviews and Meta-Analyses (PRISMA) 2020 guidelines (available in Supplementary Tables 1, 2) (22) and the guidelines of the Cochrane Handbook for Systematic Reviews of Interventions (23). The protocol was registered on the PROSPERO international prospective register of systematic reviews (CRD420251000872).

### 2.2 Eligibility criteria

Studies that assessed the effects of dietary supplements and medication among professional and pre-professional dancers from various disciplines in health and performance were eligible for inclusion. The Population, Intervention, Comparison, Outcomes and Study (PICOS) framework was followed (24).

The studies met the following inclusion criteria: (i) active male or female dancers engaged in regular dance training and/or performance at a professional or pre-professional levels. Professional dancers were defined as individuals employed or contracted to perform dance professionally, whereas pre-professional dancers were defined as students enrolled in vocational, conservatory, university, or academy-based training programs intended to prepare for a professional career; (ii) use of one or more dietary supplements or medication. For the purposes of this review, ‘medication use’ refers to the consumption of therapeutic pharmacological agents, including prescription and over-the-counter medications when used for health-related purposes, while excluding recreational substances, illicit drugs, and performance-enhancing agents; (iii) assessed prevalence or incidence of supplement and medication use and outcomes related to dance performance, athletic performance, health benefits, injury and pathology prevention or management; (iv) randomized comparisons between supplements/medication versus placebo, or an alternative supplement/medication were preferred but not required; (v) all study designs were considered eligible for inclusion.

Exclusion criteria were: (i) mixed population of dancers, combining professional and pre-professional levels or multiple dance disciplines, unless specific subgroups data by level and discipline could be extracted; (ii) substances used for purposes other than performance or treatment of dance-related pathologies, such as recreational purposes; and (iii) outcomes unrelated to dance-specific skills or activities or to the treatments of non-specific dance pathologies or injuries.

### 2.3 Information sources

The literature search was conducted in PubMed (National Center for Biotechnology Information), Scopus (Elsevier), Web of Science (Clarivate Analytics), and Cochrane Library from inception to April 2025 without date and language restrictions and was not updated prior to manuscript submission. Grey literature sources, including preprints and unpublished studies, were not included; only peer-reviewed published articles were considered.

### 2.4 Search strategy

A search strategy was performed following combination of MeSH terms using PICO approach (24): (i) population: [(danc* OR ballet OR “contemporary danc*” OR “spanish danc*” OR jazz OR lyrical OR “urban danc*” OR “break* danc*” OR “hip hop” OR “modern danc*”); (ii) intervention: ((supplement* OR diet* OR nutr* OR food) OR (drug* OR medication*))]; (iii) comparator: no use of medication or dietary supplements; (iv) outcome; (injur* OR disorder* OR patholog* OR performance).

References of articles retrieved were checked to identify relevant studies. The complete search strategy for each database is detailed in Supplementary Table 3.

### 2.5 Selection and data collection process

All records retrieved were imported to Rayyan, a web-based tool for conducting systematic reviews. Duplicate entries were manually identified and removed by one author (MVA). The screening process then proceeded in two stages: title/abstract screening and full-text screening. Two authors (MVA and APL) independently screened all records/reports at each stage, with disagreements resolved through discussion or consultation with a third author (JJMA) when necessary. During the full-text screening phase, corresponding authors were contacted via email for reports potentially eligible but lacking sufficient specificity in their data. Reports were excluded if the necessary data could not be retrieved.

Data extraction was independently performed by the same two authors (MVA and APL). Any disagreements were resolved through discussion, with input from the remaining three authors when necessary. The final data extraction structure was developed based on the target tables intended for the manuscript informed by the Cochrane Consumers and Communication Review Group’s recommendations for the collection of key information: (i) study data: author, year of publication, study design, demographic location; (ii) participants characteristics: age, gender, sample size, dance level, discipline), (iii) supplementation or pharmacotherapeutic protocol: substance, dosage, administration schedule and duration; (iv) fitness tests, health measurements and study outcomes.

A meta-analysis was not performed due to substantial heterogeneity across the included studies. This heterogeneity was observed in study populations, types of supplements and medication assessed, interventions, outcome measures, and study designs, which limited the comparability of results.

### 2.6 Quality and risk of bias assessment

Risk of bias assessment was performed by a single author (MVA) with standardized tools, tools and predefined criteria, under methodological supervision by APL. Each included study was categorized for their level of risk of bias. The Cochrane’s Risk of Bias 2 tool (RoB 2) was used for randomized intervention studies, evaluating potential bias across five domains: randomization process, deviations from intended interventions, missing data on outcomes, measurement of outcomes and selection of reported results (25). Each study was judged as “low,” “some concerns” and “high risk” of bias. The Risk Of Bias In Non-randomized Studies of Interventions (ROBINS-I) tool was used to assess non-randomized intervention studies by emulating a hypothetical randomized trial assessing seven domains: confounding, selection of participants into the study, classification of interventions, deviations from intended interventions, missing data, measurement of outcomes and selection of the reported result (26). An overall risk of bias was assigned as “low,” “moderate,” “serious” or “critical” risk of bias. For observational studies of different designs: case-reports, case–control, cross-sectional and cohort; the Joanna Briggs Institute (JBI) critical appraisal tools were used to assess risk of bias through a battery of questions for each design (27). As this tool does not follow an algorithm to determine the overall risk of bias it was determined: “low risk” if one or less questions were answered as “unclear” and the rest as “yes”; “some concerns” if there were two or three “unclear” answers or one as “unclear” and maximum one “no,” the rest answered as “yes”; and “high” risk if two or more questions were answered as “no”.

## 3 Results

### 3.1 Search results and study selection

The database search identified 8,562 records, of which 1,862 were removed as duplicates. Title and abstract screening led to 63 articles sought for retrieval. Full-texts for 16 articles were not accessible through institutional subscriptions. Attempts were made to obtain these articles by contacting the corresponding authors twice via email. After a four-week waiting period without successful access to the manuscripts, these 16 articles were excluded from the review due to the unavailability of the full-text.

**Figure 1:**
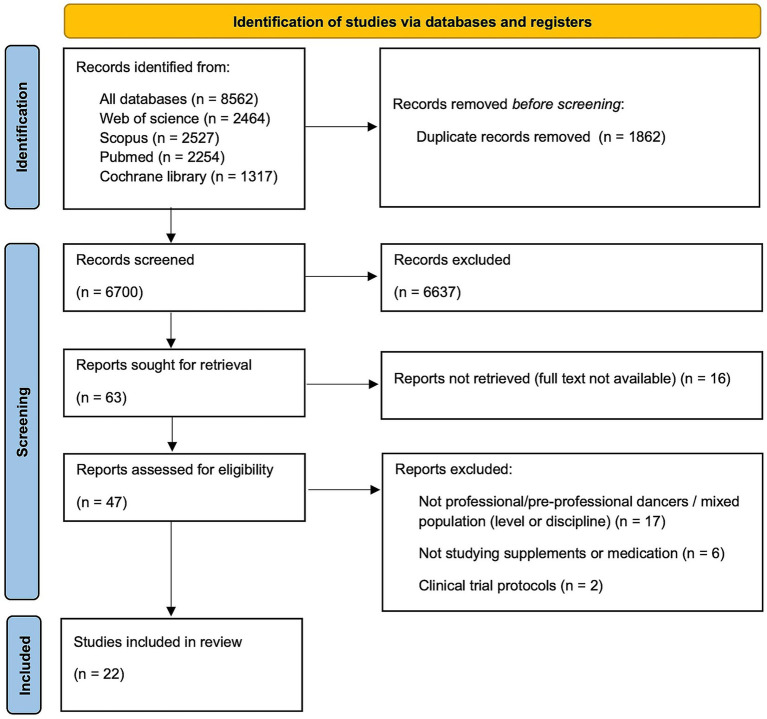
PRISMA flow diagram.

Thus, 47 full-text articles, all published in English, were assessed for eligibility: 17 reports were excluded for not matching the eligible population (e.g., amateur dancers, mixed dancer and other athletic populations), while 6 were excluded for not meeting the intervention or exposure criteria (e.g., does not include supplement or medication use) and 2 were excluded because they were clinical trial protocols. In total, 22 studies were included in the review. At the title and abstract screening stage, the percent agreement between authors was 97.0%, with a kappa coefficient of 0.855, indicating a high agreement. At the full-text screening stage, the percent agreement was 90.4%, with a kappa coefficient of 0.706, reflecting substantial agreement. Study screening and selection progress following the PRISMA flow diagram is illustrated in Figure 1.

### 3.2 Study characteristics

The population of included studies consists of 740 participants (654 female and 86 male dancers) ranging from 13 to 36 years, one study reporting a minimum age of 19 years; showing a considerable overlap in age distributions between pre-professional (13 to 30 years) and professional dancers (13 to 36 years). Ten studies investigated pre-professional dancers from different universities, conservatories and schools that include in their training program only ballet (4, 28–31), contemporary (32); or various disciplines: usually ballet and contemporary, jazz or modern (33–36). Twelve studies included professional dancers; eight from regional and international ballet companies (16, 21, 31, 37–41), one on a contemporary dance company (42), one Division I ballet dancers of the National Collegiate Athletic Association (NCAA) (43), one of hip-hop members of the Dance Sport Federation of Slovenia and International Dance Organization (44) and one of break dancers in the Spanish National Breaking Team (45). Detailed participant characteristics, including dance genre, training context, and where available training volume and experience, are reported in Table 1.

**Table 1:** Characteristics and main findings of included studies.

Study (year)	Region	Study design	Population	Intervention/exposure	Exercise tests and outcomes	Main findings	Risk of bias
Wiącek et al. (2024) (42)	Poland	Randomized, double-blind, placebo- controlled	15 female professional contemporary dancers (18–36 years)	Probiotic supplement: *Lactobacillus helveticus* (Rosell-52) and *Bifidobacterium longum* (Rosell-17) (3 × 10^9^ CFU per capsule) vs. maltodextrin and cornstarch, in the mornings for 12 weeks.	Abdominal pain (ROME IV), Pressure–pain test, Sleep quality and latency (PSQI), Fatigue (FAS), Active coping stress strategies (Mini-COPE test).	Probiotic did not significantly change abdominal pain scores (*p* = 0.48), pressure–pain thresholds (*p* = 0.58), sleep quality (*p* = 0.08), sleep latency (*p* = 0.23), fatigue (*p* = 0.30), and stress (*p* = 1.00).	Some concerns
Brooks et al. (2023) (35)	United States	Randomized, double-blind, placebo- controlled	13 female pre-professional ballet, contemporary and jazz dancers, University of Idaho dance program (20 ± 1 years)	Creatine monohydrate Creapure^®^ (0.1 g/kg/day + 0.1 g/kg/day corn-starch maltodextrin) vs. cornstarch maltodextrin (0.2 g/kg/day), both in powder form reconstituted in 0.24 L of water, during 42 consecutive days.	Body composition (DXA); Fluid cognition battery (NIHTB), psychological status (DASS); Isometric Strength (hip flexion/extension peak torque, FI); Medicine Ball Throw; Vertical Jump height; Modified Wingate (Peak power, fatigue resistance).	Creatine significantly increased body weight (58.3 ± 9.8 to 59.0 ± 10.6 kg vs. placebo: 59.4 ± 9.7 to 57.8 ± 9.0 kg; *p* = 0.010), TBW (32.2 ± 3.5 to 32.7 ± 3.6 kg vs. placebo: 32.2 ± 2.7 to 32.0 ± 3.1 kg; *p* = 0.024) and LM (39.8 ± 3.6 to 41.5 ± 4.5 kg vs. placebo: 39.8 ± 3.4 to 39.9 ± 3.6 kg; *p* = 0.020), specifically, lower appendicular LM (13.6 ± 1.5 to 14.1 ± 1.5 kg vs. placebo: 13.5 ± 1.0 to 13.5 ± 1.0 kg; *p* = 0.047). No significant improvements in performance (*p* > 0.2), cognitive (*p* > 0.2) and psychological (*p* > 0.3) outcomes were observed.	Some concerns
Alfiero et al. (2021) (33)	United States	Quasi-experimental	17 female and 1 male pre-professional ballet and modern dancers, collegiate dancers from a university (20 ± 2 years)	Whey protein isolate Dymatize Iso100^®^ 90 g vs. 40 g vs. no supplemental protein each day, during the 6-week HIIT intervention (2 HIIT sessions per week).	Body composition (DXA scan). Aerobic graded exercise test (HRpeak, VO_2_peak, VT1/VT2, LT workload, and PPO). Wingate (PPO, FI).	All groups responded similarly irrespective of protein supplementation. It significantly improved fitness parameters (*p* ≤ 0.001), except for MPPO. LM (42.7 ± 7.2 to 43.5 ± 7.3 kg; *p* < 0.05) and FM (23.4 ± 12.1 to 22.6 ± 11.9 kg; *p* < 0.05) significantly increased and decreased, respectively.	High
Brown et al. (2019) (34)	United States	Randomized, double-blind, placebo- controlled	21 female pre-professional modern and ballet dancers, School of Dance at Florida State University (18–30 years)	Whey protein isolate Dymatize Iso100^®^ 75 g (25 g 3x/day, powder form reconstituted with water first between breakfast and lunch, second between lunch and dinner, and third after dinner and before bed) vs. isocaloric maltodextrin for 12 weeks.	Body composition (DXA); RMR (indirect calorimetry); Lower body endurance (Wall sits); Lower body isometric strength (peak torque, FI); Vertical jump height; Treadmill test (VO₂max, HR); Wingate (anaerobic power, MPPO, PPO, FI).	Whey protein isolate significantly improved lean mass (70.2 ± 3.4 to 70.5 ± 3.9%; *p* < 0.05; vs. placebo: 68.8 ± 4.2 to 67.1 ± 3.8; *p* > 0.05) without changes in body weight. FM or LST were not significantly different between groups (*p* > 0.05). Neither group demonstrated changes in laboratory performance tests (*p* > 0.05).	Some concerns
Wyon et al. (2014) (37)	United Kingdom and Greece	Non-randomized controlled trial	13 female and 11 male professional ballet dancers, international touring ballet company (28 ± 5 years)	Vitamin D3 (2000 IU/day per capsule) for 4 months.	Muscular Strength (Isometric quadriceps contraction); Vertical jump externally rotated; Time-loss injury report by in-house physiotherapists.	Vitamin D3 significantly improved isometric strength (692.1 ± 363.7 to 821.9 ± 365.9 N, *p* < 0.01, η^2^ = 0.197 vs. placebo: 565.1 ± 253.1 to 566.9 ± 224.8 N, *p* > 0.05) and vertical jump height (42.8 ± 10.8 to 45.9 ± 11.2 cm, *p* < 0.01, η^2^ = 0.322 vs. placebo: 39.6 ± 7.6 cm to 38.9 ± 8.3 cm). The intervention group sustained fewer injuries than controls (5 vs. 7 injuries).	Some concerns
Wyon et al. (2019) (46)	United Kingdom	Randomized, double-blind, placebo- controlled	29 female and 38 male pre-professional ballet dancers, dance school (17–19 years)	Vitamin D3 120,000 IU (120 tablets of 1,000 IU each) vs. placebo (120 inert tablets) administered over a 4-day period.	Muscle strength (isometric mid-thigh pull); Muscular power—CMJ height, RSI; Time-loss injury report by in-house physiotherapists.	Vitamin D3 significantly improved isometric mid-thigh pull (males: 1316 ± 328 to 1,399 ± 179 N; females: 865 ± 147 to 891 ± 163 N, *p* = 0.022 vs. placebo; males: 1389 ± 261 to 1,389 ± 199 N; females: 967 ± 181 to 922.0 ± 162.7 N). Vertical jump height did not significantly change (males: 35.7 ± 5.24 to 37.3 ± 5.06 cm; females: 25.2 ± 3.63 to 25.6 ± 2.95 cm vs. placebo; male: 38.1 ± 3.07 to 38.6 ± 2.25 cm; female: 24.4 ± 4.76 to 25.9 ± 4.57 cm). Injury incidence was reduced (≥2 total injuries: 8.9 vs. 36.4%; *p* < 0.03, φc = 0.35), especially for traumatic injuries (≥2 injuries: 11.1 vs. 31.8%; *p* < 0.03, φc = 0.25), for the intervention group.	Low
Brown and Wyon (2014) (32)	Netherlands and United Kingdom	Non-randomized balanced crossover	10 female pre-professional contemporary dancers, dance school (20 ± 2 years)	MGI energy bar (47.3 g of carbohydrates, 2.1 g of fat, and 9.6 g of protein) vs. water, 15–20 min before warm up.	Blood glucose (mmol/L) Perceived pleasure-displeasure during contemporary class (Hardy and Rejeski Feeling Scale)	MGI bar consumption resulted in a gradual rise in blood glucose (F_1,114_ = 6.273; *p* = 0.014) and improved Feeling Scale scores (F_1,114_ = 9.891; *p* = 0.002), peaking at 30 min post-consumption.	High
Hoch et al. (2011) (70)	United States	Prospective cross-sectional	22 female professional ballet dancers, dance company (23 ± 5 years) 14 with abnormal FMD (<5%) 8 with normal FMD (>5%)	Folic acid (10 mg/day in the morning with food) only the group with abnormal flow-mediated dilation (<5%) for 4 weeks.	Fasting serum folate levels (μg/mL) before and after supplementation. Brachial artery (FMD) and velocity (%). Arterial pressure (mmHg) and heart rate (BPM)	Folic acid supplementation significantly improved FMD in dancers with endothelial dysfunction (2.9 ± 1.5%, to 7.1 ± 2.3%; *p* < 0.0001).	High
Mahlamäki and Mahlamäki (1988) (36)	Finland	Non-randomized controlled trial	25 female pre-professional ballet and modern dancers, music and dance high school in Kuopio (mean 17 years)	Ferrous sulphate equivalent to 100 mg elemental Fe/day for 10 weeks.	Blood Hb (g/L), iron stores (μg/L), and ferritin (μg/L) serum levels. Anemia prevalence.	Iron supplementation reduced anemia prevalence by 75%. Hemoglobin level differences between dancers and non-dancers disappeared. Participants with reduced iron stores did not change.	High
Warren et al. (2003) (39)	United States	Randomized, placebo-controlled	36 female professional ballet dancers, national and regional dance schools and companies (22 ± 5 years) 13 eumenorrheic 13 amenorrhoeic	Hormone replacement therapy: Premarin (conjugated estrogens), 0.625 mg, on days 1 to 25; and Provera (progesterone), 10 mg, on days 16 to 25, in a 30-day cycle and 1,250 mg/day, calcium citrate for 2 years.	Menstrual and hormonal profile E2, FSH, LH, PRL, DHEAS, and T levels. Eating attitudes (EAT-26). Spinal, wrist and foot bone density (Dual and Single Photon Absorptiometer)	HRT did not significantly change spine BMD (5.60 ± 1.10% vs. placebo: 4.46 ± 2.80%), wrist BMD increased less in the HRT group (0.91 ± 5.12% vs. placebo: 3.19 ± 1.48%) and foot BMD decreased (−6.49 ± 2.04% vs. placebo: 1.48 ± 2.83%). Five untreated participants resumed normal menses, without a normalization of BMD, despite significant increases in spine BMD (11.83 ± 6.53%).	Some concerns
Brenke et al. (1996) (41)	Germany	Case report	1 female professional ballet dancer (27 years)	Clinical interview.	Physical examination: muscular tone, blood pressure, HR. Blood testing. Urine volume (ml/24 h). Protein urine excretion (mg/24 h). Kidney morphology- ultrasonography.	Medication use: furosemide 20–40 mg/day, chlorthalidone 50–100 mg/day, bisacodyl 7.5 mg/day, NSAID (diclofenac and ibuprofen).	Low
Holderness et al. (1994) (40)	United States	Case–control	50 female professional ballet dancers, national and regional ballet companies (13–31 years)	EAT-26, DSM-III-R and substance use questionnaires and clinical interview: 5 point weighted scale, ranging 0 “never” -4”daily/weekly.”	Assess the frequency of use of diet pills, diuretics, laxatives, amphetamines, tranquilizers and other drugs.	Diet pills and diuretics reported very infrequent use in dancers and controls. Laxatives showed a slightly higher average, but still infrequent.	Some concerns
Torres-McGehee et al. (2021) (43)	United States	Cross-sectional	26 NCAA Division I female collegiate ballet dancers (21 ± 4 years)	Eating Disorder Inventory—3 (EDI-3) symptom checklist.	Assess the use of diet pills, laxatives, diuretics.	Prevalence of supplement use was low: laxatives 0%, diet pills 7.7% and diuretics 3.8%.	Low
Angoules et al. (2018) (30)	Greece	Descriptive retrospective cohort	46 female pre-professional ballet dancers, dance school (29 ± 5 years)	Questionnaire focusing on activity and back pain.	Attacks of low back pain, intensity of pain, absence from training, episodes seeking medical consultation and the selected intervention.	52.2% dancers with low back pain sought medical consultation. 45.7% received conservative treatment: anti-inflammatories or muscle relaxants (76.2%), rest (23.8%). Additional treatments: physiotherapy, acupuncture, manual therapy, and lumbar epidural corticoid injections.	Some concerns
Peric et al. (2016) (16)	Croatia	Cross-sectional	21 female professional ballet dancers, Croatian National Theater Ballet (17–32 years)	Questionnaire of substance use (QSU) (socio-demographic variables, consumption of substances-cigarettes, alcohol, and drugs- and amenorrhea).	Assess the use of analgesics and dietary supplements.	58% reported using a nutritional supplement; while 90% used analgesics (53% occasional; 37% regular/frequent).	Some concerns
Burckhardt et al. (2011) (28)	Switzerland	Cohort	127 female pre-professional ballet dancers, Prix de Lausanne competitors (15 to 18 years)	3-day qualitative dietary record.	Assess the use of multivitamins and only calcium supplements.	16% of dancers use an only calcium supplement, while 57% of dancers use vitamin or multi-mineral supplement, or both.	Low
Prus et al. (2019) (44)	Slovenia	Cross-sectional	100 female and 14 male professional hip-hop dancers, members of Dance Sport Federation of Slovenia and International Dance Organization (15–28 years)	Questionnaire about food supplements, general nutrition and doping [Sekulic et al. (21); Kondrič et al. (71)].	assess the use of food supplements, general nutrition and doping.	42.1% used at least one supplement. The most common being energy tabs, vitamins, and recovery drinks.	Some concerns
Civil et al. (2019) (4)	United Kingdom	Cross-sectional	20 female pre-professional ballet dancers, Royal Conservatoire of Scotland (18 ± 1 years)	Combined method of a self-reported quantitative food diary and 24-h recall interviews.	Assess the use of dietary supplements (vitamins, antioxidants, probiotics, herbal extracts, minerals, and fish oil).	11.55% of dancers reported using at least one dietary supplement.	Low
Bonbright. (1989) (29)	United States	Cross-sectional	32 female pre-professional ballet dancers (15–18 years)	Dietary record during five consecutive days.	Assess the food, fluid and nutritional supplements ingested.	34.37% of dancers used supplements, most commonly vitamin/mineral supplement supplying 100–150% of RDA of 19 essential micronutrients (15.6%) (*n* = 5). Supplementation increased the RDA of vitamins by 85.1% and minerals by 25%.	High
Wolman et al. (2013) (31)	United Kingdom and Greece	Cohort	13 females and 6 male professional ballet dancers, international touring ballet company (26 ± 9 years)	Lifestyle questionnaire.	Assess the use of vitamin B complex and vitamin C supplements.	21.05% of dancers used vitamin B complex and vitamin C supplements.	Low
Sekulic et al. (2010) (21)	Croatia	Cross-sectional	16 female and 9 male professional ballet dancers, Croatian National Theater Ballet (19 + years)	Questionnaire for studying substance use.	Consumption and habits with alcohol, cigarettes, drugs, nutritional supplements, analgesics and “doping.”	72% of dancers reported using nutritional supplements, primarily isotonic beverages (52%) and carbohydrate/recovery supplements (20%). Analgesic use was common, and 12% reported appetite suppressant use.	Some concerns
Montalbán-Méndez et al. (2023) (45)	Spain	Cross-sectional	1 female and 7 male professional breaking dancers, Spanish National Breaking Team (18–35 years)	Survey on the frequency of consumption (FFQ) of sports supplements and ergogenic aids.	Assess the use of whey protein, isotonic drink, caffeine, creatine, BCAA, glutamine, beta-alanine and multivitamins.	Supplement use included whey protein (21.43%), creatine (21.42%), caffeine (14.29%), multivitamins (14.29%), isotonic drinks (7.14%), BCAA (7.14%), glutamine (7.14%), and beta-alanine (7.14%).	Low

Data are presented in prevalence of use (%) and mean ± SD. MGI, Moderate glycemic index; DASS, Depression; Anxiety and Stress Scale; NIHTB, National Institute of Health Toolbox; FI, Fatigue index; VT, Ventilatory threshold; LT- Lactate threshold; HR, heart rate; PPO, peak power output; RMR, resting metabolic rate; LST, lean soft tissue; MPPO, mean peak power output. LM, lean mass; FM, fat mass; CMJ, Countermovement jump; RSI, Reactive Strength Index; FMD, flow mediated dilation; E2, estradiol; FSH, follicle stimulating hormone; LH, luteinizing hormone; PRL, prolactin; DHEAS, dehydroepiandrosterone sulphate; T, testosterone; BMD, bone mass density; NSAID, non-steroidal anti-inflammatory drug; EAT-26, eating attitude test, 26; DSM-III-R, diagnostic and statistical manual of mental disorders; 3er edition revised; RDA, recommended dietary allowance; BCAA, branched chain amino acid.

Among the included articles, six observational studies assessed the use of vitamin and mineral supplements (4, 28, 29, 31, 44, 45), weight control substances (laxatives, diuretics and appetite suppressants) by four studies (21, 40, 41, 43), and other ergogenic aids (amino acids, caffeine, isotonic drinks, recovery supplements, fish oil, antioxidants, herbal extracts) (4, 21, 45). Analgesic and anti-inflammatory medications were assessed by four studies (16, 21, 30, 41). Three observational studies examined weight-management substance use (40, 41, 43). The most studied supplements in interventional studies were Vitamin D3 (37, 46) and whey protein isolate (33, 34), followed by creatine (35), energy bars (32), and probiotics (42) as ergogenic supplements; and folic acid (38), ferrous sulphate (36) and hormone therapy (39) as therapeutic interventions.

To assess physical performance, the included intervention studies followed several exercise protocols and measured different outcomes, described in Table 1, along with supplementation protocols. The most frequent performance outcomes reported were vertical jump height, isometric strength (34, 35, 37, 46), anaerobic power (33–35), aerobic parameters (VO_2max_, lactate threshold) (33, 34), and fatigue (33, 34, 42). Moreover, other studies evaluated health-oriented parameters such sleep outcomes (42), mood (32, 35), mental health (35, 42), pain outcomes (42), time-loss injury incidence (37, 46), flow-mediated endothelial dilation (38), iron levels (36), bone density (39) and kidney function (41). The prevalence and frequency of use of weight control substances, dietary supplements, and analgesics were measured through questionnaires, dietary records and clinical interviews.

### 3.3 Risk of bias

The RoB 2 assessment tool was used to assess the risk of bias of included randomized controlled trials (*n* = 6) (34, 35, 39, 42, 46) and one study with a quasi-experimental design (33). The overall bias raised some concerns. Selection of the reported results and randomization process raised the most concerns as sample size was low or significantly different between the control and intervention groups and some outcome measurements were not addressed in the study’s methods (42), and the randomization and analysis methods were not clear (35, 39). Only one study reported an overall high risk of bias, because of deviations from the intended interventions and selection of the reported result, as the experiment was not blinded and the control group was not given a placebo (33).

The ROBIN-1 V2 tool was used for bias assessment for non-randomized intervention studies (*n* = 4) (32, 36–38). Overall bias raised moderate (37) to serious risk (32, 36, 38). The most concerning domains were unmeasured confounding factors and the measurement of the outcome, as assessors were probably aware of the intervention participants received. There was also one study with a serious risk of missing data due to the lack of displaying individual data results (32). Bias in the classification of interventions raised some concerns, as no information was available on the potential influence of the classification status on the results (32, 36, 38).

The JBI critical appraisal tool was used for observational studies of different designs (*n* = 12), one case-report (41), one case–control (40), seven cross-sectional (4, 16, 21, 29, 43–45) and three cohort studies (28, 30, 31). Overall risk of bias resulted low in one case-report (41), three cross-sectional (4, 43, 45) and two cohort studies (28, 31). Some concerns were noted in the case–control study due to unclear control of confounding (40), three cross-sectional studies due to unclear definitions of inclusion and exclusion criteria and confounding factors (16, 21, 44), and one cohort study also due to unclear definition of confounding (30). High risk of bias was reported in one cross-sectional study due to unclear definition of inclusion and exclusion criteria, confounding and statistical analysis methodology (29).

Overall risk of bias and traffic light plots are available as Supplementary Figures 1–3.

### 3.4 Results of synthesis

#### 3.4.1 Prevalence of dietary supplement and medication use

##### 3.4.1.1 Performance-aiding supplements

Ergogenic aids were reported in studies on ballet, breaking and hip-hop professional dancers using self-reported questionnaires or dietary records (21, 44, 45). The characteristics of each study and prevalence of dietary supplement use are summarized in Table 1. The most reported ergogenic supplements were whey protein (21.4%), creatine (21.4%), caffeine (14.3%), beta-alanine (7.1%), glutamine (7.1%), and BCAA (7.1%) (45), energy tablets, carbohydrate and recovery supplements (20%), and isotonic drinks (52%) (21, 44).

##### 3.4.1.2 Other dietary supplements

Vitamin and mineral supplement use varied widely across populations of professional and pre-professional dancers. Prevalence of supplements ranged from 57% to 11.6% in pre-professional ballet dancers, including multivitamins and multimineral and single calcium supplements (4, 28, 29); while in professional hip-hop and ballet dancers, the prevalence ranged from 21.1% to 42.1%, with multivitamins, vitamin B complex, and vitamin C among the most frequent (31, 44). Additional supplements reported included probiotics, antioxidants, fish oil and herbal extracts among pre-professional ballet dancers (4).

##### 3.4.1.3 Weight management substances

Three observational studies examined weight-management substance use among professional female ballet dancers using self-reported questionnaires and clinical reports (40, 41, 43). Overall, the use of diet pills, laxatives and diuretics was infrequent, with reported prevalences in a collegiate cohort being 0% in laxatives, 3.8% for diuretics, and 7.7% in diet pills (40, 43).

In contrast, severe misuse has been reported in isolated instances. One case-report described a 27-year-old professional ballet dancer using diuretics (furosemide, chlorthalidone), and laxatives (bisacodyl) alongside NSAIDs, resulting in renal dysfunction and papillary necrosis with calcification (41).

##### 3.4.1.4 Medication

Four studies reported medication, specifically for musculoskeletal pain in ballet dancers. Across a pre-professional cohort, 45.7% of dancers who sought medical care for low back pain, received primarily conservative treatment with short courses of anti-inflammatory or muscle relaxants (76.2%) or rest (24%), and in some cases physiotherapy, acupuncture, manual therapy and epidural corticosteroids (30). Among professional Croatian ballet dancers, analgesic prevalence was 90%, either occasionally (53%) or regularly (37%) (16), while in a previous study from the same company, frequency of use ranged from rare (56%) to often (16%) or regular (12%) (21).

#### 3.4.2 Performance-focused outcomes

The following narrative provides the findings in performance and health outcomes of the included intervention studies. Full statistical results, measures and effect size estimates where available, are reported in Table 1.

##### 3.4.2.1 Vitamin D

Two studies investigated the effects of vitamin D3 supplementation on performance and injury (37, 46). One study in professional ballet dancers reported increased isometric quadriceps contraction and vertical jump height, with 2,000 IU/day for four months, with moderate to large effects on both strength and jump height, while no meaningful changes were observed in the control group for strength and a decrease in muscle power. Fewer time-loss injuries were reported among dancers receiving vitamin D3 than among controls, despite a larger sample size in the intervention group (37).

Additionally, a loading dose of vitamin D3 (120,000 IU administered over 4 days) in pre-professional ballet dancers also showed an small to moderate improvement in isometric strength, compared with minimal changes in placebo; however, no significant effects were observed in muscular power or height during countermovement jump. The intervention group had significantly fewer participants compared to the placebo group, particularly for traumatic injuries, representing a moderate positive association between supplementation and reduced injury risk (46).

In both studies, the prevalence of vitamin D insufficiency (<75 nmol/L) in dancers was high. In one study the baseline prevalence was 87%, reducing this number to 36% after the loading dose at the end of the intervention (46), while in the other study, all dancers had been identified with vitamin D insufficiency the previous year to the intervention (37).

##### 3.4.2.2 Whey protein isolate

Two studies investigated the effects of whey protein isolate supplementation on performance and body composition in pre-professional ballet and modern dancers (33, 34). One study examined the effects of 90 g/day, 40 g/day or no supplemental protein during a 6-week high-intensity interval training (HIIT) (33). Cardiorespiratory fitness (i.e., VO_2peak_) and short-term, high-intensity anaerobic performance (i.e., Wingate test) significantly improved in all groups independent of supplementation. Similarly, improvements were found in body composition with increases in lean mass and decreases in fat mass in the supplemented groups and no change in the control group without significant differences.

Conversely, in another study in which 75 g/day of whey protein isolate (25 g three times daily) was compared with a placebo for 12 weeks in female dancers (34). Significant improvements in lean body mass were observed in the intervention group, without changes in fat mass, compared with a slight increase in the placebo group. No significant between-group differences were found in cardiorespiratory fitness, muscular isokinetic strength, vertical jump height, and Wingate test outcomes.

##### 3.4.2.3 Creatine

The effects of 0.1 g/kg/day of creatine supplementation for 42 days in female pre-professional ballet, contemporary and jazz dancers showed significant changes in body composition (35). Supplementation resulted in increases in lean mass, lower appendicular lean mass, and total body water, with a parallel increase in body mass in the intervention group. Despite body composition changes, no significant differences were found between creatine and placebo in performance outcomes [isokinetic hip strength, anaerobic PPO, medicine ball throw and vertical jump height, cognitive and psychological assessments (DASS)].

#### 3.4.3 Health-related outcomes

##### 3.4.3.1 Moderate glycemic index energy bar

One study investigated the effects of a moderate glycemic index (MGI) energy bar compared to water on blood glucose and perceived pleasure-displeasure during contemporary dance class in female pre-professional dancers (32). The ingestion of the MGI bar resulted in a gradual rise in blood glucose, peaking at 30 min post-consumption. The Feeling Scale showed significant changes over time, with MGI consumption producing higher scores that peaked at 30 min and decreased by 60 min.

##### 3.4.3.2 Probiotics

Supplementation with *Lactobacillus helveticus* (Rosell-52) and *Bifidobacterium longum* (Rosell-17) (3 × 10^9^ CFU, daily for 12 weeks) showed no significant effects on quality of life outcomes compared with placebo in female professional contemporary dancers (42). No significant between-group differences were observed for abdominal pain scores, pressure–pain thresholds, sleep quality and latency, fatigue and active coping stress strategies.

##### 3.4.3.3 Micronutrient therapy (folic acid and iron)

Two intervention studies examined the effects of micronutrient supplementation on vascular and hematological parameters in female dancers (36, 38). One study investigated the effect of folic acid supplementation on professional ballet dancers, only dancers with abnormal flow-mediated dilation (FMD < 5%) were supplemented with 10 mg/day of folic acid for 4 weeks (38). Dancers with endothelial dysfunction showed an improvement in flow-mediated dilation following supplementation, with a statistically significant increase compared to baseline, while heart rate, mean arterial pressure and peak brachial artery diameter remained unchanged.

Another study assessed the effects of iron supplementation (100 mg Fe/day) for 10 weeks in pre-professional ballet and modern dancers (36). Baseline data showed that low hemoglobin was more prevalent in dancers than controls, with 15% exhibiting iron-deficiency anemia (low Hb, low serum iron and ferritin, increased transferrin), while no cases occurred in the control subjects. Iron supplementation reduced hemoglobin levels by 75% and eliminated the significant difference in hemoglobin levels between the treated and untreated groups; however, it did not restore iron stores to normal levels (serum ferritin >30 ng/mL).

##### 3.4.3.4 Hormone therapy replacement

One study investigated the effects of hormone replacement therapy (HRT) combined with calcium supplementation on bone mineral density (BMD) and reproductive hormones in female professional ballet dancers (39). The intervention included conjugated estrogens on days 1–25, progesterone on days 16–25 of a 30-day cycle, and daily calcium for 2 years. At baseline, amenorrheic dancers had lower BMD at the spine, wrist and foot compared with eumenorrheic dancers and controls. After 24 months, spine BMD in treated amenorrhoeic dancers increased compared with placebo, wrist BMD increased less in the HRT group vs. placebo, and foot BMD decreased in the intervention group compared to an increase in placebo. Five dancers experienced a return or onset of normal menses, all being in the placebo group, with a significant increase in spine BMD, although values did not normalize relative to controls. Hormonal profiles in these dancers were consistent with hypothalamic amenorrhea.

## 4 Discussion

### 4.1 Performance outcomes

Our findings indicate that prevalence data, primarily derived from observational and survey-based studies, reported use of dietary supplements among studies was variable, differing between professional dancers (21–42%) and pre-professional (12–34%) cohorts, including whey protein, creatine, caffeine, multivitamins, vitamin B complex, and vitamin C as the commonly consumed. Despite widespread supplement use, included observational studies showed variability in methodological quality, ranging from low risk of bias to some concerns and, in one case, high risk of bias; therefore, prevalence estimates should be interpreted with caution, as they may be influenced by study-level bias and small sample sizes.

Evidence regarding the effects of supplementation on performance outcomes presents variable effects on key performance outcomes, derived from randomized and non-randomized controlled trials. Vitamin D supplementation has been shown to yield improvements in isometric strength (≈18.7%) and vertical jump height (≈7.1%) in elite ballet dancers following a four-month oral regimen of 2,000 IU/day compared with control (37). Moreover, a RCT in adolescent dancers reported a 7.8% increase in muscle strength and reduced traumatic injury incidence with vitamin D supplementation (46). Recent data suggest that these effects are likely mediated through its role in skeletal muscle repair, regeneration, and muscle function via activation of the vitamin D receptor (VDR) within skeletal muscle (47). These improvements align with recent meta-analysis suggesting enhanced strength and power metrics in athletic populations, particularly among individuals with insufficient baseline levels (48, 49). These mechanisms may be particularly relevant, particularly in ballet, given the high neuromuscular demands associated with repeated jumps, landings, and rapid directional changes. Furthermore, the observed reduction in injury incidence following supplementation may reflect improved muscle function and recovery capacity, potentially contributing to enhanced movement control during high-impact dance-specific tasks (47). These data suggests a possible role of targeted nutritional interventions to influence muscular strength performance parameters, potentially relevant to dancers.

Conversely, limited intervention studies in dancers have not consistently demonstrated performance benefits for other commonly used supplements. Whey protein supplementation in female collegiate dancers showed no significant changes in aerobic and anaerobic fitness, or body composition compared with placebo. Small changes in lean soft tissue and fat mass were observed after 6 weeks combined with HIIT, or after 12 weeks with supplementation alone; although, both studies involved small samples and the latter may not have targeted high-intensity anaerobic performance (33, 34). These findings suggest that merely increasing macronutrient intake, in the absence of a documented deficiency or targeted training stimulus, may not translate into performance gains, and that the intervention duration may have been insufficient to elicit significant changes in body composition.

Creatine supplementation in female dancers increased lean mass and total body water, consistent with evidence in athletic populations showing enhanced fat-free mass and cellular hydration through elevated intramuscular phosphocreatine availability (50, 51). However, unlike well-documented improvements in strength, power and anaerobic performance typically observed when combined with resistance or high-intensity training, in the trial included in this review (35), no gains were observed in isokinetic strength, anaerobic power, or jump performance. This discrepancy likely reflects the absence of targeted strength training in the studied samples, an essential stimulus for creatine’s ergogenic effects, and may also be related to evidence indicating that performance responses to creatine in active females are still inconsistent (52).

Thus, dietary supplements such as creatine, caffeine, and beta-alanine are relatively common among the included dancer populations (44, 45); however, despite their well-documented ergogenic properties in athletes, their effects remain largely unexplored in dance settings. Existing evidence indicates these supplements may contribute to maintain or improve physical and cognitive performance during rehearsals and performances (53). Future studies should explore the ergogenic potential of these dietary supplements in dancers.

In addition, available data reports a high prevalence of use of analgesic and anti-inflammatories, whereas other pharmacological groups have not yet been investigated regarding prevalence, performance or health outcomes in dancers. The reported prevalence in up to ~90% of in a cohort of ballet dancers raises concerns that symptom suppression, rather than true performance enhancement, may contribute to maintained output under fatigue or pain conditions. Although the available evidence is derived mainly from cross-sectional and cohort designs, these studies are subject to limitations in confounding control and measurement consistency, which may affect the robustness and comparability of prevalence estimates. Evidence suggests that analgesic and anti-inflammatory medication, particularly paracetamol, may contribute to improvements in acute exercise endurance (e.g., time-trial and cycling in heat) (54, 55) and neuromuscular performance in specific settings (e.g., repeated sprint and repeated maximal voluntary contraction) (54, 56), whereas the existence of ergogenic effects of NSAIDs on sports performance remains inconclusive (57). Despite inhibiting cyclooxygenase (COX) activity and thereby reducing the acute anabolic response to resistance exercise (58, 59) they appear to affect long-term training adaptations in muscle strength and hypertrophy, especially at high doses of ibuprofen (60, 61).

Although adverse effects were not assessed across the included studies, potential risks may arise not only from the physiological effects of supplements but also from inappropriate use patterns, including excessive dosing, product combinations without consideration of cumulative intake, and use outside recommended protocols (53). For example, gastrointestinal and cardiovascular side effects linked to NSAID, alongside the potential interference with training adaptations (60, 61). Similarly, stimulant use, including caffeine, may contribute to nausea, anxiety, accelerated heart rate, and sleep disturbances, particularly when consumed in high doses (53). These considerations highlight the importance of interpreting supplementation outcomes within a broader context of the dancer’s health, rather than solely in terms of ergogenic benefits.

Overall, the available evidence base in dancers is characterized by a predominance of observational and small-scale interventional studies, predominantly female participants from classical ballet and contemporary dance backgrounds, with limited high-quality randomized controlled trials. Taken together, the performance outcomes indicate that while some supplementation (e.g., vitamin D) appears to be beneficial, the heterogeneity in study designs, that presented concerning risk of bias, dosage, and outcome measures limits the possibility to establish firm conclusions regarding broad ergogenic or functional performance improvements in dancers.

### 4.2 Health-related outcomes

Beyond performance, health-related outcomes associated with substances used by dancers require careful consideration. Survey data indicate that 48% to 72% of dancers across 53 countries regularly use dietary supplements, principally multivitamins, caffeine and vitamin C, with fatigue reduction and immunity support as primary motives (12, 13). However, over-the-counter analgesics are also prevalent (e.g., up to a 90% in a professional female ballet dancer cohort) (16, 21). Studies in elite athletes consistently suggest that NSAID use rates are concerning about medication misuse, high dosing, suppression of symptoms and potential masking of underlying conditions (6, 62). Although, these findings cannot be directly extrapolated to dancer populations, they indicate a potential need to further research patterns of use and associated risks in dancers. The health-related outcomes reflect a dual scenario: the potential benefits of targeted supplementation (e.g., correcting a deficiency), as well as risks associated with unmonitored or excessive use and underlying nutritional or recovery deficits. This also applies to analgesic and anti-inflammatory medications, which can be effective for managing acute pain, but their benefits diminish when treating chronic injuries (63).

Health-related findings in dancers suggest a complex interplay between micronutrient status, energy availability, musculoskeletal health, injury risk, and pharmacological or supplemental interventions. High rates of vitamin D insufficiency (94%) were observed among young dancers from a sunny country, values correlated with serum ferritin and indoor training exposure (64). A systematic review of ballet dancers (five studies: two interventional, three observational) suggests that vitamin D status is associated with reduced injury occurrence and improved muscle function, but evidence remains limited (14). Given vitamin D’s role in bone mineralization, neuromuscular function and immune modulation, the implications are substantial for dancers, who frequently train indoors, perform repetitive load activities and have periods of heavy rehearsal.

Regarding the consistent pattern of high prevalence of low serum 25-hydroxyvitamin D (25(OH)D) (64) this aligns with findings of low serum ferritin levels in female elite ballet dancers, ~68% below athletic norms (<50 ng/mL) (65, 66). Given iron’s central role in oxidative metabolism and oxygen transport, deficiencies may compromise health (e.g., anemia, fatigue) and performance. Nutritional monitoring is particularly relevant in aesthetic disciplines such as dance, where energy availability may be constrained (66, 67). In a cohort of 40 elite ballet dancers (22 female, 18 male; 19–38 years), 41% had minimal to depleted ferritin (<50 ng/mL), with females at greater risk (65). Iron deficiency (ID) is exacerbated by aesthetic pressures, high training volumes, and low energy availability (LEA), impairing oxygen transport, mitochondrial function, and fatigue resistance (36, 67). The nutritional consequences of dietary restraint among young female dancers not only compromise health (e.g., predispose to anemia, reduced immune function, impaired cognition) but also undermine performance and increase injury risk.

Beyond micronutrients, dancers are at risk of Relative Energy Deficiency in Sport (RED-S), which encompasses LEA, endocrine dysfunction, impaired bone health, immunosuppression, and cardiovascular alterations (68). Interventions such as hormone replacement therapy or folic acid supplementation may provide partial benefit, but appear insufficient to normalize clinical parameters (38, 39). While large-scale studies in dancers are sparse, monitoring of professional cohorts has identified low thyroid T3, elevated cortisol, suppressed sex hormones and bone health markers in female dancers with risk-scores for LEA (69). The practical consequence is that health-related outcomes in dancers are not purely about being able to train and perform but also being able to do so in a sustainable manner while maintaining health. For example, persistent vitamin D or iron deficiencies may increase injury incidence, bone stress problems, delay recovery, and ultimately curtail careers. This suggests that supplementation and pharmacologic interventions may be framed not only as ergogenic or therapeutic, but as integral to dancer health monitoring and long-term welfare.

It is important to recognize that supplement use is already highly prevalent across the included studies, while clinically relevant deficiencies seem to remain common in the included dancer populations. This suggests that further research should assess if supplementation practices are optimally aligned with individual needs or underlying contributors (e.g., insufficient dietary intake, limited sun exposure, or low energy availability). Consequently, a more targeted approach to supplementation strategies in dancers may be beneficial, guided by screening and biochemical assessment, and clear indications. A more targeted, needs-based approach would minimize unnecessary intake, reduce masking unresolved health issues and enhance the effectiveness of interventions intended to restore or maintain health.

### 4.3 Methodological considerations, gaps in the literature and future research

While research on dancers’ use of supplements and medications is growing, methodological limitations impede definitive conclusions. A potential limitation of this review is the exclusion of studies for which full-text articles could not be retrieved. This may have introduced a data availability bias, as the excluded studies could not be assessed for eligibility or included in the synthesis. In addition, the risk of bias assessment was conducted by a single reviewer rather than in duplicate, as generally recommended. This approach may increase the possibility of subjective judgment influencing the assessment; however, the use of standardized tools, predefined criteria, and methodological supervision was intended to enhance consistency and minimize potential bias. A key limitation of the current evidence base is its restricted population coverage. Most included studies involved predominantly female dancers from classical ballet and contemporary disciplines, which limits the generalizability of findings to the wider dancer population and non-classical genres. Finally, the heterogeneity among studies prevented quantitative synthesis, limiting the possibility of generating pooled estimates.

Furthermore, many studies rely on cross-sectional or survey designs, which limit causal inference and introduce self-report bias. Prevalence surveys (e.g., more than 90% in one UK dancer sample) (13) provide descriptive data but cannot establish efficacy or safety. Additionally, heterogeneity in intervention protocols (dosage, duration, participant training status, discipline, outcome measures) limits comparability. For example, protein supplementation in dancers yielded null findings in body composition despite presumed ergogenic effects (34). Many performance studies include mixed athletic samples, limiting dancer-specific relevance. Consequently, well-powered RCTs in dancers with clearly defined performance endpoints, safety monitoring, especially for medications, high-dosage supplementation, and long-term effects. Future research should evaluate dose–response relationships, training interactions, sex differences, and the effects of energy availability and menstrual dysfunction (RED-S) on outcomes.

### 4.4 Practical applications

From a practical standpoint, the findings suggest actionable recommendations for practitioners working with dancers. Routine screening of vitamin D and iron status is advisable, particularly during periods of high training load or indoor training environments, as supplementation aimed at correcting clinically relevant deficiencies may support bone and muscle health, recovery, and injury prevention. In this context, targeted vitamin D supplementation (e.g., 2,000 IU/day) may be beneficial in dancers with vitamin D insufficiency to improve strength, jump performance and injury prevention. In contrast, the current evidence for ergogenic benefits of supplementation in healthy or nutritionally sufficient dancers remains less consistent. Supplements such as whey protein should not be assumed to improve performance or composition unless coupled with appropriate training and nutritional optimization; therefore, individualized nutrition plans and performance monitoring remain essential. Medication use, including analgesics and anti-inflammatories should be closely monitored, and educational programs implemented to address risks of self-medication, injury masking and, and impaired recovery. Finally, dance organizations should integrate interdisciplinary health support programs (nutrition, medical oversight, psychological support) to ensure safe and effective use of supplements and medications, aligning with performance goals and short, medium and long-term health. In doing so, the dance sector can adopt evidence-informed practice rather than relying on anecdotal or normative approaches.

## 5 Conclusion

This systematic review of pharmacological and dietary supplements use among professional and pre-professional dancers reveals a widespread but heterogeneous practice, measured performance benefits, and tangible health implications. Strategic supplementation, notably vitamin D, showed measurable improvements in strength, jump performance and injury incidence, though results vary across studies. Health-related outcomes are equally significant: high rates of vitamin D, iron deficiency, and low energy availability place dancers at elevated risk of injury, fatigue, and compromised health-span. Despite prevalent nutritional supplementation, these risks, persist and may contribute to the widespread analgesic and anti-inflammatory use. Methodological heterogeneity, small sample sizes, and concerns regarding risk of bias limit the certainty of the evidence. As a result, findings from lower-quality studies may have a disproportionate influence on the overall narrative synthesis, preventing firm conclusions and limiting the strength of recommendations. Nevertheless, the evidence supports routine screening of micronutrient status, energy-availability, alongside informed, clinically supervised supplementation. Integrating nutritional and medical oversight should be considered essential to performance preparation rather than optional. Future research should prioritize well-designed dancer-specific RCTs, with clearly defined performance, health, and injury outcomes, and an evaluation of efficacy and safety. Only then evidence-based guidelines can be developed to support the unique physiological demands and health vulnerabilities of dancers.

## Data Availability

The original contributions presented in the study are included in the article/Supplementary material, further inquiries can be directed to the corresponding author.
